# The Preoperative Peripheral Blood Monocyte Count Is Associated with Liver Metastasis and Overall Survival in Colorectal Cancer Patients

**DOI:** 10.1371/journal.pone.0157486

**Published:** 2016-06-29

**Authors:** Shidong Hu, Zhenyu Zou, Hao Li, Guijun Zou, Zhao Li, Jian Xu, Lingde Wang, Xiaohui Du

**Affiliations:** 1 Department of General Surgery, Chinese People’s Liberation Army General Hospital, Beijing, China; 2 Department of General Surgery, Chinese People’s Liberation Army Air Force General Hospital, Beijing, China; 3 Department of General Surgery, Chinese People’s Liberation Army 474 Hospital, Wulumuqi, Xinjiang, China; The Ohio State University, UNITED STATES

## Abstract

**Background:**

Colorectal cancer (CRC) is the third most common malignancy in males and the second most common in females worldwide. Distant metastases have a strong negative impact on the prognosis of CRC patients. The most common site of CRC metastases is the liver. Both disease progression and metastasis have been related to the patient’s peripheral blood monocyte count. We therefore performed a case-control study to assess the relationship between the preoperative peripheral blood monocyte count and colorectal liver metastases (CRLM).

**Methods:**

Clinical data from 117 patients with colon cancer and 93 with rectal cancer who were admitted to the Chinese People’s Liberation Army General Hospital (Beijing, China) between December 2003 and May 2015 were analysed retrospectively, with the permission of both the patients and the hospital.

**Results:**

Preoperative peripheral blood monocyte counts, the T and N classifications of the primary tumour and its primary site differed significantly between the two groups (P < 0.001, P < 0.001, P = 0.002, P < 0.001), whereas there were no differences in the sex, age, degree of tumour differentiation or largest tumour diameter. Lymph node metastasis and a high preoperative peripheral blood monocyte count were independent risk factors for liver metastasis (OR: 2.178, 95%CI: 1.148~4.134, P = 0.017; OR: 12.422, 95%CI: 5.076~30.398, P < 0.001), although the risk was lower in patients with rectal versus colon cancer (OR: 0.078, 95%CI: 0.020~0.309, P < 0.001). Primary tumour site (P<0.001), degree of tumour differentiation (P = 0.009), T, N and M classifications, TNM staging and preoperative monocyte counts (P<0.001) were associated with the 5-year overall survival (OS) of CRC patients. A preoperative peripheral blood monocyte count > 0.505 × 10^9^ cells/L, high T classification and liver metastasis were independent risk factors for 5-year OS (RR: 2.737, 95% CI: 1.573~ 4.764, P <0.001; RR: 2.687, 95%CI: 1.498~4.820, P = 0.001; RR: 4.928, 95%CI: 2.871~8.457, P < 0.001).

**Conclusions:**

The demonstrated association between preoperative peripheral blood monocyte count and liver metastasis in patients with CRC recommends the former as a useful predictor of postoperative prognosis in CRC patients.

## Introduction

According to the latest data from GLOBOCAN cancer statistics, colorectal cancer (CRC) is the third most common malignancy in males and the second most common in females, responsible for an estimated 693,900 deaths in 2012 worldwide [1]. Colorectal liver metastases (CRLM) are detected in 20–25% of patients at initial presentation but will develop in another 40–50% following primary tumour removal. The most common site for CRC metastases is the liver[2,3]. The 5-year overall survival (OS) of patients with resectable CRLM is > 40% but < 10% in those with unresectable CRLM[4]. There are currently no criteria allowing clinicians to identify the patients most likely to develop CRLM, such that approximately 90% of patients are unable to undergo curative surgery at the time of diagnosis[5]. Therefore, the identification of predictors of CRLM would be of immense benefit, enabling early treatment tailored to the individual risk of developing metastases.

Previous studies have identified a relationship between the peripheral blood monocyte count and the immune status of cancer patients. The peripheral blood monocyte count includes the number of regulatory dendritic cells (DCs), which contribute to immune suppression in cancer patients by activating and promoting the differentiation of regulatory CD4+CD25+ T cells. Thus, cancer patients with abnormally high peripheral blood monocyte counts have a poor prognosis, [6,7] and both tumour progression and metastasis have been linked to the degree of immune suppression[8]. However, the relationship between preoperative peripheral blood monocyte counts and CRLM was not clear. Therefore, in this retrospective study we analysed the clinical data of patients with CRC and CRLM to determine whether the preoperative peripheral blood monocyte count is related to the development of CRLM and/or the prognosis of patients with CRC.

## Methods

### Inclusion and exclusion criteria

The inclusion criteria were (1) hospitalization at the Chinese PLA General Hospital and radical surgery performed by surgeons above deputy chief physician status; (2) diagnosis of CRC based on the postoperative pathology and the guidelines published in the 7^th^ edition of the American Joint Committee on Cancer Staging Manual and (3) no previous radiotherapy or chemotherapy as confirmed by medical history when obtaining preoperative peripheral blood monocyte count. Patients with acute or chronic infection, immune system diseases, or multiple primary malignancies or those who were undergoing emergency surgery were excluded. All enrolled CRC patients were staged according to the Union for International Cancer Control (UICC) tumor-node-metastasis (TNM) classification system.

### Clinical data

From December 2003 to May 2015, clinical data were collected from 238 patients with CRC who underwent radical surgery and were diagnosed by postoperative pathology at the Department of Surgery, Chinese People's Liberation Army General Hospital (Beijing, China). 33 patients with initial metastatic disease were selected for operation owning preoperative imaging with resectable metastatic disease (18 patients achieved R0 resection). A final cohort of 210 patients was analyzed after the exclusion of 28 patients with missing preoperative peripheral blood monocyte count (n = 19), or missing integrated medical records (n = 6), or having immune system diseases (n = 3). The mean (±SD) age of the 137 male and 73 female patients was 56.1 (±12.9) years (range: 24–87 years). Colon cancer was diagnosed in 117 patients and rectal cancer in 93 patients. Within this group, there were 27 patients with well-differentiated, 124 with moderately differentiated and 59 with poorly differentiated cancer. The largest tumour diameter ranged from 0.8 to 18.0 cm (4.9±2.3 cm). Four patients had stage T1, 26 had stage T2, 104 stage T3 and 76 stage T4 disease. N0 was determined in 108 patients, N1 in 62 patients and N2 in 40 patients. A status of M0 was confirmed in 177 patients and M1 in 33 synchronous CRLM patients (including one with both liver and lung metastases). TNM stage 1 was diagnosed in 22 patients, stage 2 in 72 patients, stage 3 in 83 patients and stage 4 in 33 patients. The mean preoperative peripheral blood monocyte count was 0.53 (±0.21) × 109/L (range 0.14–1.98 × 109/L). Liver metastases were diagnosed based on preoperative imaging findings and postoperative pathology. Whole blood was collected into ethylene diamine tetraacetic acid containing blood collection tubes. Preoperative peripheral blood monocyte counts were performed at the clinical laboratory, Chinese People's Liberation Army General Hospital, using standard procedures on a Sysmex XS-1000i automated haematology analyser. The preoperative peripheral blood monocyte count was acquired from blood tests performed routinely before surgery. There were 127 patients received adjuvant radiochemo- or chemotherapy including: 18 TNM stage 2 patients with poorly differentiated cancer, 83 TNM stage 3 patients, 26 patients of 33 TNM stage 4 patients (5 TNM stage 4 patients could not receive adjuvant radiochemo- or chemotherapy and 2 TNM stage 4 patients refused to accept adjuvant radiochemo- or chemotherapy). Any additional information obtained from medical records. The patients were then divided according to the presence (CRLM) or absence (CRC) of liver metastasis. All patients provided written informed consent to participate in the study, which was approved by the ethics committees of the Chinese People's Liberation Army General Hospital, Beijing, China.

### Follow-up

Patient follow-up was conducted by a combination of phone calls and letters. The follow-up period ended on July 1, 2015. OS was defined as the time from the completion of surgery to the follow-up date or the death of the patient. The median follow-up period was 28 months.

### Statistical analysis

Statistical analyses were performed using IBM SPSS 17.0 (SPSS Inc., Chicago IL, USA). Quantitative variables are presented as the mean ± standard deviation. Measurement data with a normal distribution and homogeneity of variance were analysed for statistical differences using Student’s t test; otherwise, the Wilcoxon rank test was used. A χ^2^ test was used to assess differences in the numerical data and the Wilcoxon rank test for differences in relative levels. The influence of the preoperative peripheral blood monocyte count and other clinicopathological factors on the development of liver metastases from CRC was determined using multivariate logistic regression models. Receiver operating characteristic (ROC) curves were plotted to calculate the area under the ROC curve (AUC). The optimal cut-off value for the monocyte count as a prognostic indicator was calculated using the Youden index (YI). The influence of clinical and pathological factors on survival was evaluated using the Kaplan-Meier method followed by a log-rank test of the statistical differences found. A Cox regression model was used in the multivariate prognostic analysis. P < 0.05 was considered to indicate statistical significance.

## Results

### The relationship between the clinicopathological characteristics of the patients and liver metastasis

In a univariate analysis (Table 1), there were no statistically significant differences between patients with (CRLM group) and without (CRC group) metastases with respect to sex, age, degree of tumour differentiation and the largest tumour diameter. By contrast, the preoperative peripheral blood monocyte count, T classification, N classification and primary site of the tumour significantly differed between the two groups (P < 0.05).

**Table 1:** Relationship between colorectal cancer liver metastases and clinicopathological characteristics.

Clinicopathological Characteristics	Without liver metastasis n = 177	Liver metastasis n = 33	P value
Age (years)	55.53±12.89	59.21±12.63	0.13
Gender			0.83
Male	116	21	
Female	61	12	
Monocyte (10^9^/L)	0.48±0.15	0.80±0.29	<0.001
Primary site			<0.001
Colon	88	29	
Rectum	89	4	
Tumor diameter (cm)	4.924±2.383	4.485±1.779	0.41
Differentiation			0.54
Well	22	5	
Moderately	104	20	
Poorly	51	8	
T classification			0.002
T1	4	0	
T2	24	2	
T3	93	11	
T4	56	20	
N classification			<0.001
N0	100	8	
N1	50	12	
N2	27	13	

### Logistic regression analysis of risk factors for liver metastases in CRC

Multivariate logistic regression analysis (Table 2) showed that positive lymph nodes and a high preoperative peripheral blood monocyte count were independent risk factors for liver metastasis in patients with CRC (OR: 2.178, 95%CI: 1.148~4.134, P = 0.02; OR: 12.422, 95%CI: 5.076~30.398, P < 0.001).The risk of liver metastases was significantly lower in patients with rectal cancer than in those with colon cancer (OR: 0.078, 95%CI: 0.020~0.309, P < 0.001).

**Table 2:** Logistic regression analysis of risk factors for liver metastases.

Variable	B	S.E.	Wald	df	Exp(B) (95%CI)	P value
N classfication N0 vs. N1/N2	0.778	0.327	5.670	1	2.178 (1.148–4.134)	0.02
Monocyte^a^	2.519	0.457	30.448	1	12.422 (5.076–30.398)	< 0.001
Primary site Colon vs. Rectum	-2.550	0.702	13.179	1	0.078 (1.148–4.134)	< 0.001
Constant	-6.600	1.497	19.439		0.001	< 0.001

^a^ Patients were divided into four groups according to the preoperative peripheral blood monocyte counts: < 0.30×109/L, 0.30< <0.50×109/L, <0.70×109/L, >0.70×109/L.

### ROC curve for the optimal cut-off value and AUC

**Fig 1:**
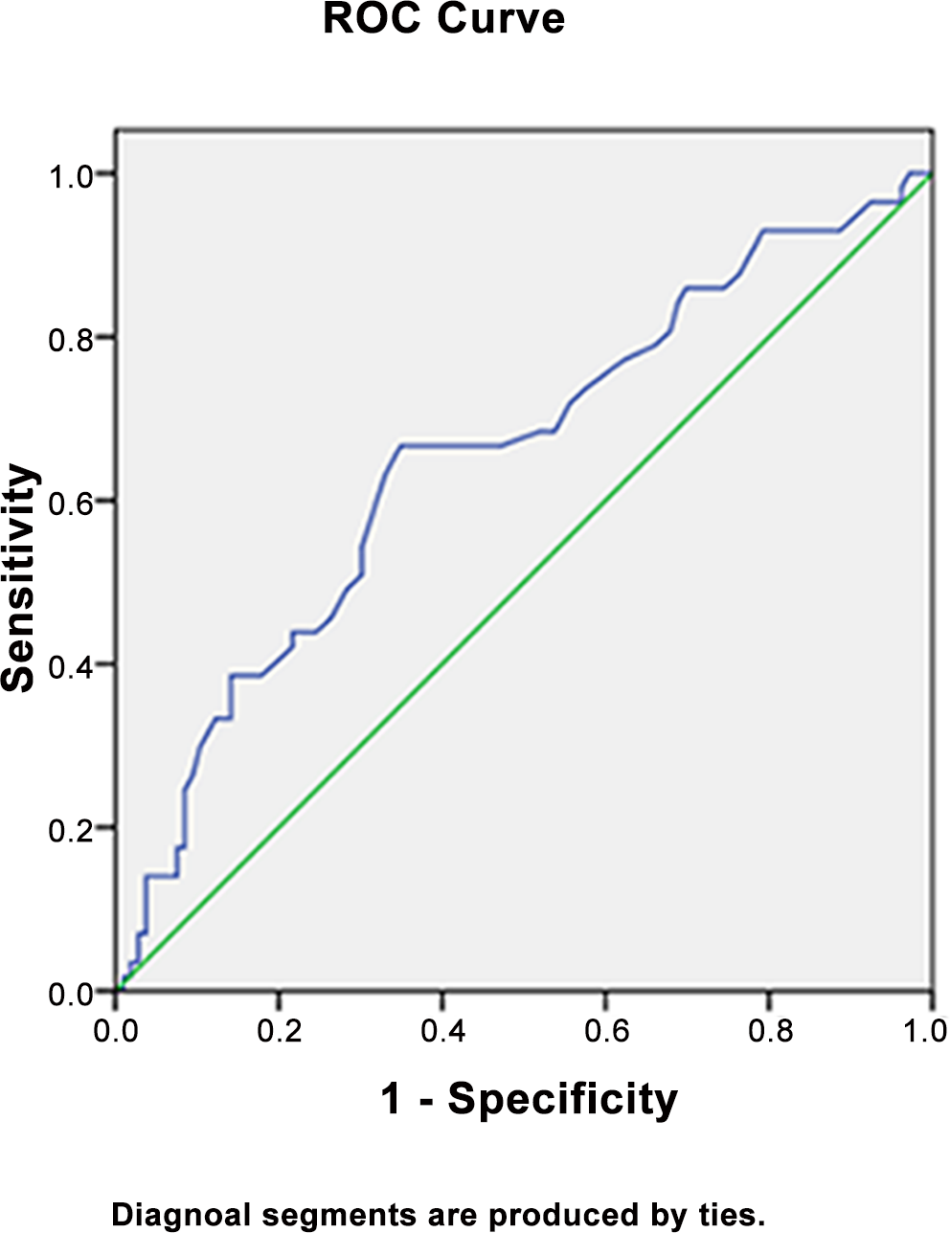
ROC curve for preoperative monocyte count. The ROC curve for preoperative monocyte count is represented by the line chart with an AUC of 0.653 (95% CI: 0.563~0.742, *P* = .001).

A monocyte count 0.505 ≥ 10^9^/L, and thus a maximum YI = 0.318, resulted in an optimal cut-off value of 0.505 × 10^9^ cells/L. The AUC was 0.653. The patients were therefore divided into those with low (< 0.505 × 10^9^/L) and high (≥ 0.505 × 10^9^/L) monocyte counts (Fig 1).

### Association of the clinicopathological characteristics with 5-year OS

**Fig 2:**
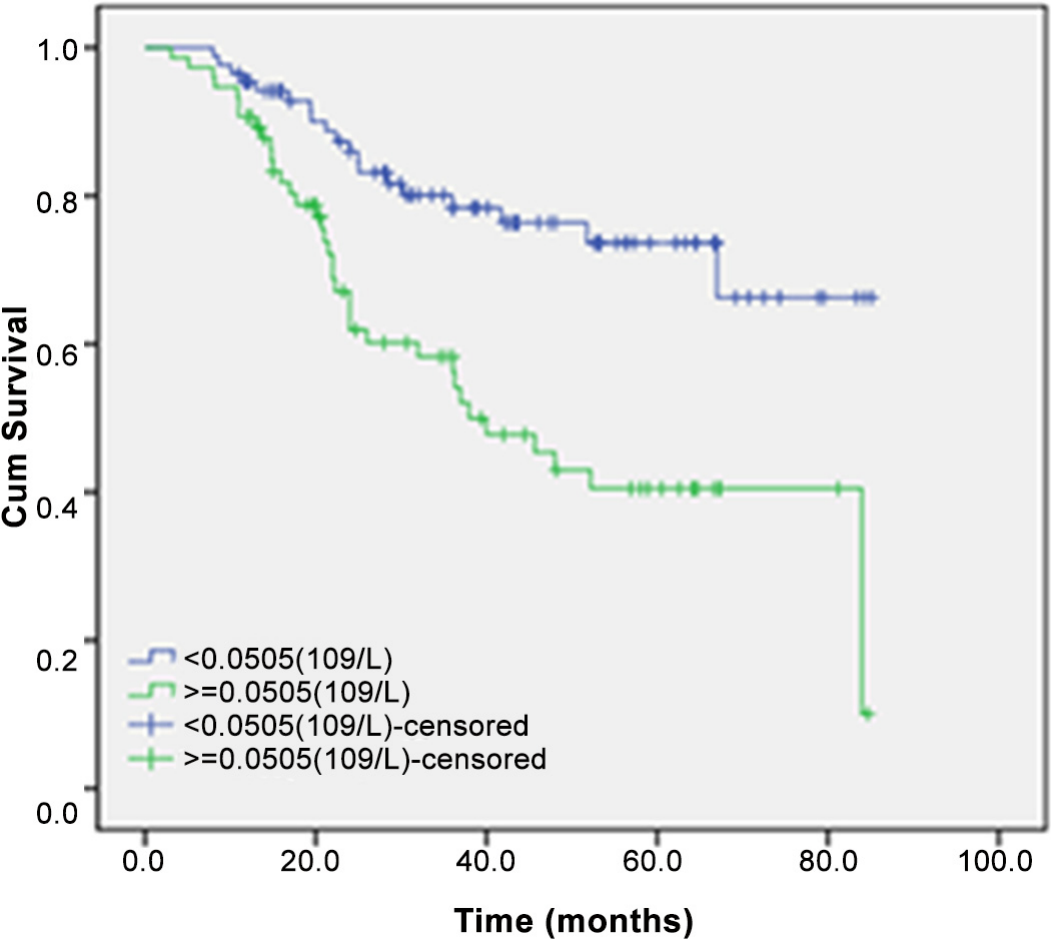
OS curve grouped by preoperative monocyte count. Patients with high preoperative monocyte count (≥ 0.505) had a significantly poorer OS than those with low preoperative monocyte count (< 0.505) (*P* < .001).

Univariate analyses showed that primary tumour site (P < 0.001), degree of tumour differentiation (*P* = 0.009), T, N and M classifications, TNM staging and preoperative peripheral blood monocyte count (*P* < 0.001) were associated with the 5-year OS rate (Table 3). Besides, patients with a high preoperative peripheral blood monocyte count, as defined above, had a significantly poorer 5-year OS than those with a low preoperative peripheral blood monocyte count (40.3% vs. 73.6%; *P* < 0.001) (Fig 2).

**Table 3:** Univariate analysis for prognosis in 210 patients with colorectal cancer.

Clinicopathological Characteristics	N	5-years OS rate(%)	P value
Age (years)			0.12
< 60	133	64.0	
≥ 60	77	47.3	
Gender			0.32
Male	137	62.3	
Female	73	44.1	
Monocyte			< 0.001
< 0.505(10^9^/L)	111	73.6	
≥ 0.505(10^9^/L)	99	40.3	
Primary site			< 0.001
Colon	117	47.2	
Rectum	93	72.4	
Size of largest tumor diameter			0.65
< 50mm	108	53.7	
≥ 50mm	102	60.9	
Differentiation			0.009
Well	27	68.3	
Moderately	124	61.3	
Poorly	59	37.0	
T classification			< 0.001
T1+T2	30	85.6	
T3	104	77.2	
T4	76	23.9	
N classification			< 0.001
N0	108	77.0	
N1	62	52.2	
N2	40	17.0	
M classification			< 0.001
M0	177	70.2	
M1	33	9.3	
TNM staging			< 0.001
I	22	88.9	
II	72	87.5	
III	83	52.7	
IV	33	9.3	

### Multivariate analyses of prognostic factors

Using the prognostic factors from the univariate analyses and the clinically significant factors, we performed a Cox regression analysis. The preoperative peripheral blood monocyte count, primary tumour site, degree of tumour differentiation, T, N and M classifications and TNM staging were included. The results showed that the preoperative peripheral blood monocyte count (≥ 0.505 × 10^9^/L), high T classification and liver metastasis were independent risk factors for the 5-year OS of CRC patients (RR: 2.737, 95% CI: 1.573~4.764, P < 0.001; RR: 2.687, 95%CI: 1.498~4.820, P = 0.001; RR: 4.928, 95%CI: 2.871~8.457, P < 0.001) (Table 4).

**Table 4:** Cox regression analysis for prognosis in 210 patients with colorectal cancer.

Clinicopathological Characteristics	Relative risk (95% CI)	P value
Monocyte	2.737 (1.573–4.764)	< 0.001
Primary site	0.590 (0.284–1.222)	0.16
Differentiation	1.279 (0.793~2.064)	0.31
T classification	2.687 (1.498~4.820)	0.001
N classification	1.335 (0.875~2.037)	0.18
M classification	4.928 (2.871~8.457)	< 0.001
TNM staging	2.368 (0.946~5.929)	0.07

## Discussion

The presence of liver metastases had a strong negative impact on the prognosis of CRC patients. Currently, there are no effective early predictors of CRLM, and the mechanism of CRLM development is unclear. The risk factors that favour CRLM have been evaluated in several studies. Hur et al. measured low expression levels of let-7i microRNA (miR) and high expression levels of miR-10b in the primary tumour tissue of CRLM patients[9]. A high level of miR-885-5p expression in serum was associated with an increased likelihood of developing CRLM [odds ratio (OR): 5.5, 95% confidence interval (CI): 1.1–26.8, *P* = 0.03; OR: 4.9, 95% CI: 1.2–19.7, *P* = 0.02; OR: 3.1, 95% CI: 1.0–10.0, *P* = 0.05], while, the discovery was based on tissue and serum through intensive validation[9]. Wang et al. showed that the high level of serum miR-29a expression was a risk factor for liver metastases in patients with CRC[10]. Cheng et al. suggested that the increased plasma expression of miR-141 favoured metastasis and poor survival in colon cancer patients, morever, the study was conducted in two independent cohorts consisting of two different ethnic populations providing compelling evidence, while, detecting small RNA was difficult and cost highly[11]. Recent studies have demonstrated the close relationship between tumour metastasis and epithelial-mesenchymal transition (EMT). Several transcription factors, including zeb1, zeb2, slug, twist, snail and members of the miR-200 family, promote EMT by altering the expression of downstream genes, including β-catenin, placental cadherin (P-cad), epithelial cadherin (E-cad) and matrix metalloproteinase[12]. In the study of Chen et al., the decreased expression of E-cad contributed to CRLM[13]. Sun et al. reported that patients with colon cancer characterised by high P-cad expression were at an increased risk of developing liver metastases. The proposed mechanism was based on the suppression of E-cad expression by P-cad, which also promoted β-catenin expression[14].

The immunosuppressive environment established by the tumour allows its further growth and metastasis. A previous study showed a close relationship between the peripheral blood monocyte count, including DCs, and the immune response to the tumour[15]. In peripheral tissues, DCs exist as immature cells; their eventual maturation requires stimulation by cytokines and antigens. These activated DCs play an important role in antigen presentation and in the anti-tumour immune response of antigen-specific cytotoxic lymphocytes[16,17]. Other researchers have reported that regulatory DCs may suppress the proliferation and activation of CD4+CD25- and CD8+CD25- T cells, resulting in immune suppression and thus inhibition of an immune attack on tumour cells[7]. Wilcox et al. proposed that haematopoietic and inflammatory cytokines within the tumour microenvironment promote monocyte proliferation, which might not only drive the suppression of host anti-tumour immunity but also promote tumour angiogenesis and perhaps even tumour growth[18]. In our study, CRLM patients had a significantly higher preoperative peripheral blood monocyte count than that of CRC patients (0.80±0.29 ×10^9^/L vs. 0.48±0.15 ×10^9^/L, P<0.001) while, there were no statistically significant differences in sex, age, degree of tumour differentiation and the largest tumour diameter. Logistic regression analysis showed that the preoperative peripheral blood monocyte count was an independent risk factor for the development of liver metastases in CRC. There were a close relationship between CRLM and preoperative monocyte count. Taken together, these observations suggest that the high counts in CRC patients are due to an increase in regulatory DCs, leading to an immunosuppressive state that favours metastasis of the primary tumour.

There is increasing evidence of an association between the peripheral blood monocyte count and prognosis in cancer patients. Sasaki et al. retrospectively analysed the relationship between the preoperative peripheral blood monocyte count and clinicopathological factors or long-term prognosis in 198 patients with hepatocellular carcinoma treated by curative resection. They demonstrated a significantly worse 5-year disease-free survival in patients with a preoperative peripheral blood monocyte count > 300/mm^3^ (14.8%) than in those with a count ≤ 300 cells/mm^3^ (14.8% vs. 29.2%). A preoperative peripheral blood monocyte count > 300 cells/mm^3^ was identified as an independent risk factor for a disease-free survival of < 5 years[19]. Gustafson et al. examined changes in the peripheral blood of 373 patients with clear renal cell carcinoma. They found that the level of CD14^+^HLA^-^DRlo/neg monocytes in the peripheral blood of these patients correlated with the intensity of CD14 staining in tumours and adversely affected survival[20]. Marcheselli et al. analysed data from 428 patients with follicular lymphoma who were enrolled in a randomized clinical trial (FOLL05). They showed that patients with a peripheral blood absolute monocyte count (AMC) > 0.63 × 10^9^/L had a poorer 5-year progression-free survival (PFS) than that of patients with a peripheral blood AMC ≤0.63 × 10^9^/L (44% vs. 61%, *P* = 0.001)[21]. In the study of Tadmor et al. of 1450 patients with classical Hodgkinֹ’s lymphoma (cHL), those with an AMC > 750/mm^3^ had poorer 10-year PFS and 10-year OS than those of cHL patients with an AMC ≤ 750/mm^3^ (65% vs. 81%, *P* < 0.001 and 78% vs. 88%, *P* = 0.01, respectively). In a multivariate analysis, the AMC was determined to be of prognostic significance for PFS [hazard ratio (HR), 1.54, *P* = 0.006] and OS in patients with nodular sclerosis diagnosed by histology (HR, 1.54, *P* = 0.006; HR, 1.56; *P* = 0.04) [22]. In the present study, 5-year OS was significantly poorer in patients with a high (≥ 0.505 × 10^9^/L) versus low (< 0.505 × 10^9^/L) preoperative peripheral blood monocyte count. In fact, a preoperative peripheral blood monocyte count (≥ 0.505 × 10^9^/L) was identified as an independent risk factor for 5-year OS in CRC patients. An elevated preoperative peripheral blood monocyte count might reflect a high degree of immune suppression and high levels of inflammatory cytokines. The latter are important in many cellular processes, including the development of malignancies, and thus may further augment the inflammatory response[23]. Immune suppression together with the nonspecific inflammatory response could have a negative impact on the 5-year OS of CRC patients.

In conclusion, our study was able to demonstrate an association between the preoperative peripheral blood monocyte count and the presence of liver metastasis in patients with CRC. Thus, the preoperative peripheral blood monocyte count may be an inexpensive and feasible approach to predict the postoperative prognosis of these patients. However, the limitations of this study were the single-centre retrospective design and the small sample size. Larger prospective studies are needed to confirm our results and to identify the exact mechanisms linking an increase in the preoperative peripheral blood monocyte count with CRLM before it can be used as a prognostic indicator in CRC patients.

## Supporting Information

S1 Checklist STROBE checklist. (DOCX)

S1 File Clinical data from medical records and follow up. (PDF)

## Abbreviations

CRC: colorectal cancer
CRLM: colorectal liver metastases
DCs: dendritic cells
ROC: receiver operating characteristic
AUC: area under the receiver operating characteristic curve
YI: Youden index
EMT: epithelial-mesenchymal transition
MMP: matrix metalloproteinase
CRCC: clear renal cell carcinoma
FL: follicular lymphoma
PFS: progression-free survival
OS: overall survival
cHL: classical Hodgkin Lymphoma
AMC: absolute monocyte count
CI: confidence interval
HR: hazard ratio
OR: odds ratio
