# Supplementary material for: The Preoperative Peripheral Blood Monocyte Count Is Associated with Liver Metastasis and Overall Survival in Colorectal Cancer Patients
Source: PLoS One. 2016 Jun 29;11(6):e0157486. doi: 10.1371/journal.pone.0157486 (PMC4927165; doi:10.1371/journal.pone.0157486)
Supplement: S1 File — (PDF) [file pone.0157486.s002.pdf]

| bianhao | Gender | Age | Site | Different | Diameter | T | N | M |
|---|---|---|---|---|---|---|---|---|
| 1 | 2 | 83 | 1 | 2 | 11 | 4 | 0 | 0 |
| 2 | 1 | 71 | 2 | 3 | 8 | 4 | 1 | 0 |
| 3 | 1 | 72 | 2 | 2 | 3 | 4 | 1 | 1 |
| 4 | 1 | 77 | 1 | 3 | 4.5 | 3 | 1 | 0 |
| 5 | 2 | 76 | 1 | 2 | 2 | 3 | 0 | 0 |
| 6 | 1 | 75 | 2 | 1 | 3 | 2 | 1 | 0 |
| 7 | 1 | 56 | 2 | 2 | 1.5 | 4 | 0 | 0 |
| 8 | 2 | 61 | 1 | 1 | 2.5 | 4 | 0 | 0 |
| 9 | 1 | 58 | 1 | 2 | 5 | 3 | 0 | 0 |
| 10 | 1 | 78 | 1 | 3 | 3.5 | 3 | 0 | 0 |
| 11 | 1 | 72 | 1 | 2 | 5 | 3 | 1 | 0 |
| 12 | 2 | 77 | 1 | 2 | 3.5 | 3 | 0 | 1 |
| 13 | 2 | 45 | 1 | 1 | 8 | 4 | 2 | 0 |
| 14 | 1 | 44 | 2 | 2 | 5 | 2 | 0 | 0 |
| 15 | 1 | 55 | 1 | 3 | 5 | 3 | 0 | 0 |
| 16 | 2 | 51 | 2 | 2 | 3.5 | 2 | 0 | 0 |
| 17 | 2 | 68 | 2 | 2 | 4 | 3 | 1 | 0 |
| 18 | 2 | 42 | 1 | 3 | 11 | 4 | 2 | 0 |
| 19 | 1 | 68 | 1 | 2 | 10 | 3 | 0 | 0 |
| 20 | 2 | 46 | 2 | 2 | 3.5 | 3 | 0 | 0 |
| 21 | 2 | 51 | 2 | 2 | 3.5 | 3 | 0 | 0 |
| 22 | 2 | 80 | 2 | 1 | 4.5 | 3 | 2 | 0 |
| 23 | 2 | 49 | 1 | 3 | 4.5 | 4 | 1 | 0 |
| 24 | 1 | 43 | 2 | 1 | 4 | 4 | 0 | 0 |
| 25 | 2 | 72 | 2 | 2 | 5 | 3 | 1 | 0 |
| 26 | 1 | 54 | 2 | 1 | 4 | 3 | 1 | 0 |
| 27 | 1 | 54 | 2 | 2 | 5.5 | 3 | 1 | 0 |
| 28 | 1 | 54 | 2 | 2 | 3 | 1 | 1 | 0 |
| 29 | 1 | 60 | 1 | 3 | 18 | 4 | 1 | 0 |
| 30 | 1 | 46 | 1 | 2 | 6 | 3 | 0 | 0 |
| 31 | 1 | 60 | 2 | 1 | 5 | 3 | 0 | 0 |
| 32 | 1 | 60 | 1 | 3 | 5.5 | 3 | 1 | 0 |
| 33 | 2 | 53 | 2 | 2 | 4 | 2 | 1 | 0 |
| 34 | 2 | 42 | 1 | 2 | 2 | 3 | 0 | 0 |
| 35 | 1 | 53 | 2 | 2 | 7 | 3 | 0 | 0 |
| 36 | 2 | 44 | 1 | 2 | 3.5 | 3 | 2 | 0 |
| 37 | 1 | 57 | 1 | 3 | 7 | 4 | 2 | 0 |
| 38 | 1 | 34 | 2 | 1 | 2.5 | 3 | 1 | 0 |
| 39 | 1 | 53 | 2 | 2 | 3 | 3 | 2 | 0 |
| 40 | 1 | 39 | 2 | 2 | 6.5 | 3 | 0 | 0 |
| 41 | 1 | 32 | 1 | 3 | 10 | 4 | 0 | 0 |
| 42 | 2 | 66 | 2 | 2 | 5 | 3 | 2 | 0 |
| 43 | 2 | 54 | 2 | 1 | 7 | 3 | 1 | 0 |
| 44 | 1 | 69 | 1 | 2 | 3 | 3 | 1 | 0 |
| 45 | 1 | 33 | 2 | 3 | 6 | 4 | 0 | 0 |
| 46 | 1 | 64 | 2 | 1 | 6 | 3 | 0 | 0 |
| 47 | 2 | 49 | 2 | 2 | 3 | 4 | 0 | 0 |
| 48 | 1 | 32 | 1 | 2 | 7 | 3 | 0 | 0 |
| 49 | 2 | 62 | 1 | 1 | 5.5 | 3 | 0 | 0 |
| 50 | 2 | 61 | 1 | 2 | 1.6 | 3 | 0 | 0 |
| 51 | 2 | 47 | 1 | 1 | 2.5 | 2 | 2 | 1 |
| 52 | 1 | 45 | 1 | 3 | 8 | 4 | 0 | 0 |
| 53 | 1 | 52 | 2 | 2 | 4 | 3 | 0 | 0 |

| | | | | | | | | |
|---|---|---|---|---|---|---|---|---|
| 54 | 2 | 51 | 2 | 2 | 5 | 3 | 0 | 0 |
| 55 | 2 | 52 | 1 | 2 | 2.5 | 3 | 0 | 0 |
| 56 | 2 | 67 | 2 | 2 | 3.5 | 2 | 0 | 0 |
| 57 | 1 | 63 | 1 | 2 | 6 | 3 | 0 | 0 |
| 58 | 1 | 62 | 2 | 2 | 5 | 3 | 1 | 0 |
| 59 | 1 | 64 | 1 | 3 | 6.5 | 4 | 0 | 0 |
| 60 | 1 | 53 | 2 | 2 | 2.5 | 2 | 0 | 0 |
| 61 | 1 | 59 | 1 | 2 | 12 | 3 | 1 | 0 |
| 62 | 1 | 56 | 2 | 3 | 3.5 | 3 | 0 | 0 |
| 63 | 1 | 32 | 2 | 2 | 3.5 | 4 | 2 | 0 |
| 64 | 2 | 69 | 1 | 3 | 7 | 4 | 0 | 0 |
| 65 | 2 | 57 | 2 | 2 | 3.5 | 3 | 2 | 1 |
| 66 | 1 | 78 | 1 | 3 | 4 | 3 | 2 | 0 |
| 67 | 2 | 57 | 1 | 3 | 5 | 4 | 2 | 0 |
| 68 | 1 | 53 | 1 | 2 | 3 | 3 | 0 | 1 |
| 69 | 1 | 43 | 1 | 2 | 5 | 3 | 0 | 0 |
| 70 | 1 | 55 | 1 | 2 | 5 | 3 | 1 | 0 |
| 71 | 1 | 40 | 1 | 2 | 10 | 4 | 0 | 0 |
| 72 | 1 | 48 | 2 | 2 | 5 | 4 | 0 | 0 |
| 73 | 1 | 55 | 1 | 1 | 5 | 3 | 0 | 0 |
| 74 | 1 | 73 | 2 | 2 | 4 | 3 | 2 | 0 |
| 75 | 1 | 60 | 1 | 2 | 6 | 4 | 0 | 0 |
| 76 | 1 | 65 | 1 | 1 | 4 | 4 | 0 | 1 |
| 77 | 2 | 49 | 1 | 1 | 5 | 2 | 0 | 0 |
| 78 | 1 | 45 | 1 | 3 | 8 | 4 | 2 | 0 |
| 79 | 1 | 43 | 1 | 3 | 4 | 4 | 1 | 0 |
| 80 | 1 | 36 | 2 | 1 | 3 | 1 | 0 | 0 |
| 81 | 1 | 30 | 1 | 2 | 3 | 4 | 1 | 1 |
| 82 | 1 | 39 | 1 | 1 | 5 | 2 | 0 | 0 |
| 83 | 2 | 47 | 1 | 2 | 4 | 3 | 1 | 0 |
| 84 | 2 | 70 | 1 | 2 | 6 | 4 | 2 | 1 |
| 85 | 1 | 38 | 1 | 3 | 8 | 3 | 0 | 0 |
| 86 | 1 | 51 | 1 | 2 | 5 | 4 | 0 | 0 |
| 87 | 1 | 68 | 1 | 2 | 5 | 3 | 2 | 1 |
| 88 | 1 | 58 | 2 | 2 | 8 | 3 | 0 | 0 |
| 89 | 1 | 45 | 1 | 2 | 3 | 3 | 1 | 1 |
| 90 | 1 | 55 | 2 | 3 | 3 | 2 | 1 | 0 |
| 91 | 1 | 53 | 2 | 2 | 3 | 2 | 0 | 0 |
| 92 | 2 | 24 | 1 | 3 | 5 | 3 | 1 | 0 |
| 93 | 2 | 74 | 1 | 3 | 5.5 | 3 | 0 | 0 |
| 94 | 1 | 44 | 1 | 1 | 2.5 | 4 | 1 | 1 |
| 95 | 1 | 56 | 1 | 3 | 5 | 3 | 0 | 0 |
| 96 | 1 | 81 | 1 | 2 | 5 | 3 | 1 | 1 |
| 97 | 1 | 36 | 1 | 3 | 7 | 4 | 0 | 0 |
| 98 | 1 | 34 | 1 | 3 | 3.5 | 3 | 0 | 0 |
| 99 | 1 | 51 | 2 | 2 | 5 | 3 | 0 | 0 |
| 100 | 2 | 43 | 1 | 3 | 6 | 3 | 0 | 1 |
| 101 | 1 | 54 | 1 | 3 | 4 | 4 | 2 | 1 |
| 102 | 1 | 76 | 2 | 2 | 10 | 3 | 1 | 0 |
| 103 | 1 | 46 | 2 | 2 | 2 | 2 | 0 | 0 |
| 104 | 1 | 66 | 1 | 3 | 5 | 4 | 0 | 0 |
| 105 | 1 | 61 | 2 | 3 | 2 | 3 | 1 | 1 |
| 106 | 1 | 59 | 2 | 2 | 5 | 3 | 0 | 0 |
| 107 | 1 | 56 | 2 | 2 | 5.5 | 3 | 1 | 0 |

| | | | | | | | | |
|---|---|---|---|---|---|---|---|---|
| 108 | 2 | 45 | 2 | 2 | 1 | 2 | 0 | 0 |
| 109 | 1 | 53 | 2 | 2 | 4.5 | 3 | 1 | 0 |
| 110 | 1 | 46 | 2 | 2 | 4 | 3 | 1 | 0 |
| 111 | 2 | 32 | 2 | 3 | 5 | 3 | 0 | 0 |
| 112 | 2 | 73 | 2 | 2 | 6 | 4 | 1 | 0 |
| 113 | 1 | 55 | 2 | 2 | 5.5 | 4 | 0 | 0 |
| 114 | 2 | 66 | 1 | 2 | 4 | 4 | 2 | 0 |
| 115 | 1 | 63 | 2 | 2 | 5 | 2 | 0 | 0 |
| 116 | 1 | 56 | 1 | 3 | 10 | 3 | 1 | 0 |
| 117 | 1 | 53 | 2 | 2 | 8.5 | 4 | 0 | 0 |
| 118 | 1 | 70 | 1 | 2 | 4.5 | 4 | 2 | 1 |
| 119 | 1 | 58 | 2 | 3 | 2 | 3 | 0 | 0 |
| 120 | 1 | 53 | 2 | 2 | 4.5 | 3 | 0 | 0 |
| 121 | 2 | 75 | 2 | 3 | 3.5 | 3 | 1 | 0 |
| 122 | 2 | 60 | 2 | 2 | 4 | 3 | 0 | 0 |
| 123 | 1 | 38 | 2 | 1 | 2.5 | 4 | 0 | 0 |
| 124 | 1 | 68 | 2 | 2 | 5 | 3 | 1 | 0 |
| 125 | 2 | 43 | 1 | 3 | 4.5 | 4 | 2 | 0 |
| 126 | 2 | 59 | 1 | 2 | 3 | 3 | 1 | 0 |
| 127 | 1 | 64 | 1 | 2 | 5 | 4 | 2 | 1 |
| 128 | 1 | 51 | 1 | 2 | 4 | 4 | 1 | 1 |
| 129 | 2 | 64 | 1 | 2 | 6 | 3 | 1 | 0 |
| 130 | 1 | 44 | 2 | 2 | 3 | 2 | 0 | 0 |
| 131 | 1 | 46 | 2 | 3 | 4 | 3 | 2 | 0 |
| 132 | 1 | 55 | 1 | 3 | 3 | 3 | 1 | 0 |
| 133 | 2 | 53 | 2 | 2 | 2 | 2 | 0 | 0 |
| 134 | 1 | 63 | 2 | 2 | 8 | 3 | 0 | 0 |
| 135 | 2 | 47 | 2 | 2 | 3.5 | 2 | 2 | 0 |
| 136 | 2 | 60 | 2 | 2 | 9 | 3 | 0 | 0 |
| 137 | 2 | 68 | 1 | 3 | 3 | 4 | 1 | 0 |
| 138 | 1 | 77 | 2 | 2 | 4.8 | 3 | 1 | 0 |
| 139 | 2 | 65 | 2 | 2 | 3.8 | 4 | 0 | 0 |
| 140 | 1 | 59 | 2 | 2 | 3 | 3 | 0 | 0 |
| 141 | 1 | 64 | 1 | 2 | 2.2 | 2 | 2 | 0 |
| 142 | 2 | 87 | 1 | 2 | 7 | 3 | 0 | 0 |
| 143 | 1 | 55 | 2 | 2 | 6 | 3 | 1 | 0 |
| 144 | 2 | 42 | 2 | 1 | 4.5 | 3 | 1 | 0 |
| 145 | 1 | 46 | 2 | 2 | 3 | 2 | 0 | 0 |
| 146 | 2 | 38 | 2 | 3 | 3.5 | 4 | 2 | 0 |
| 147 | 1 | 57 | 2 | 2 | 6 | 3 | 0 | 0 |
| 148 | 1 | 41 | 1 | 2 | 4.5 | 3 | 1 | 0 |
| 149 | 1 | 51 | 2 | 1 | 0.8 | 2 | 0 | 0 |
| 150 | 1 | 41 | 1 | 2 | 8 | 3 | 0 | 0 |
| 151 | 2 | 76 | 2 | 2 | 5 | 4 | 1 | 0 |
| 152 | 2 | 57 | 1 | 2 | 5 | 3 | 0 | 1 |
| 153 | 1 | 72 | 1 | 1 | 5 | 3 | 0 | 0 |
| 154 | 1 | 30 | 1 | 2 | 5.5 | 4 | 0 | 0 |
| 155 | 1 | 54 | 2 | 3 | 4.5 | 3 | 2 | 0 |
| 156 | 1 | 56 | 1 | 2 | 3 | 2 | 0 | 0 |
| 157 | 2 | 58 | 1 | 3 | 2.7 | 4 | 2 | 0 |
| 158 | 2 | 49 | 2 | 2 | 2 | 2 | 0 | 0 |
| 159 | 2 | 51 | 1 | 2 | 5.5 | 4 | 2 | 1 |
| 160 | 2 | 68 | 1 | 3 | 7.2 | 4 | 1 | 0 |
| 161 | 1 | 52 | 2 | 2 | 3.5 | 3 | 2 | 0 |

| | | | | | | | | |
|---|---|---|---|---|---|---|---|---|
| 162 | 2 | 39 | 1 | 1 | 3.5 | 3 | 1 | 0 |
| 163 | 1 | 45 | 1 | 1 | 10 | 3 | 0 | 1 |
| 164 | 1 | 70 | 2 | 3 | 5 | 4 | 1 | 0 |
| 165 | 1 | 54 | 1 | 2 | 4 | 4 | 1 | 1 |
| 166 | 1 | 47 | 1 | 3 | 1 | 1 | 0 | 0 |
| 167 | 1 | 46 | 2 | 2 | 3.5 | 3 | 0 | 0 |
| 168 | 1 | 74 | 1 | 2 | 3 | 2 | 0 | 0 |
| 169 | 1 | 53 | 2 | 1 | 4 | 1 | 1 | 0 |
| 170 | 1 | 72 | 1 | 2 | 2.2 | 3 | 0 | 0 |
| 171 | 2 | 76 | 1 | 2 | 4.5 | 3 | 0 | 0 |
| 172 | 1 | 81 | 1 | 3 | 4 | 4 | 2 | 1 |
| 173 | 1 | 64 | 2 | 3 | 5 | 2 | 0 | 0 |
| 174 | 1 | 57 | 2 | 2 | 4 | 3 | 1 | 0 |
| 175 | 2 | 60 | 1 | 3 | 6.4 | 3 | 1 | 0 |
| 176 | 1 | 56 | 1 | 2 | 5 | 4 | 0 | 1 |
| 177 | 1 | 44 | 1 | 2 | 4 | 4 | 2 | 0 |
| 178 | 1 | 59 | 2 | 2 | 5.5 | 3 | 2 | 0 |
| 179 | 2 | 43 | 2 | 3 | 4.5 | 4 | 0 | 0 |
| 180 | 1 | 50 | 2 | 1 | 5 | 4 | 1 | 0 |
| 181 | 1 | 48 | 1 | 1 | 7 | 4 | 2 | 1 |
| 182 | 1 | 51 | 1 | 2 | 3.5 | 4 | 2 | 1 |
| 183 | 2 | 63 | 1 | 2 | 2.5 | 3 | 0 | 0 |
| 184 | 2 | 39 | 1 | 2 | 4 | 4 | 0 | 0 |
| 185 | 2 | 58 | 1 | 3 | 4 | 3 | 1 | 0 |
| 186 | 2 | 53 | 1 | 2 | 2 | 3 | 0 | 0 |
| 187 | 2 | 44 | 2 | 3 | 5 | 4 | 2 | 0 |
| 188 | 1 | 64 | 1 | 3 | 7 | 4 | 0 | 0 |
| 189 | 1 | 48 | 2 | 2 | 4 | 3 | 0 | 0 |
| 190 | 2 | 66 | 1 | 2 | 1.5 | 2 | 0 | 1 |
| 191 | 1 | 32 | 2 | 3 | 2.2 | 2 | 0 | 0 |
| 192 | 1 | 51 | 2 | 2 | 2.5 | 3 | 0 | 0 |
| 193 | 1 | 77 | 1 | 2 | 5 | 4 | 2 | 1 |
| 194 | 2 | 51 | 2 | 2 | 5 | 3 | 1 | 1 |
| 195 | 1 | 58 | 1 | 3 | 5 | 4 | 2 | 1 |
| 196 | 1 | 30 | 1 | 2 | 11 | 4 | 0 | 0 |
| 197 | 2 | 42 | 1 | 2 | 3.3 | 4 | 0 | 0 |
| 198 | 2 | 67 | 1 | 3 | 3 | 4 | 1 | 1 |
| 199 | 1 | 58 | 1 | 2 | 12 | 4 | 1 | 0 |
| 200 | 2 | 81 | 1 | 3 | 7 | 4 | 1 | 1 |
| 201 | 2 | 59 | 1 | 3 | 8 | 4 | 1 | 1 |
| 202 | 2 | 48 | 1 | 2 | 5.5 | 4 | 0 | 0 |
| 203 | 1 | 81 | 1 | 3 | 6 | 4 | 2 | 0 |
| 204 | 1 | 83 | 1 | 2 | 6 | 3 | 1 | 0 |
| 205 | 1 | 79 | 2 | 3 | 6.4 | 4 | 2 | 0 |
| 206 | 1 | 69 | 1 | 2 | 5 | 4 | 0 | 0 |
| 207 | 1 | 62 | 1 | 2 | 4 | 4 | 0 | 0 |
| 208 | 1 | 74 | 1 | 2 | 5.5 | 4 | 2 | 0 |
| 209 | 1 | 74 | 1 | 2 | 2.5 | 3 | 0 | 0 |
| 210 | 1 | 56 | 1 | 3 | 4 | 4 | 0 | 0 |

| Stage | Monocyte | OS | state |
|---|---|---|---|
| 2 | 0.021 | | |
| 3 | 0.064 | 52.2 | 1 |
| 4 | 0.089 | 45.7 | 1 |
| 3 | 0.043 | 46.1 | 0 |
| 2 | 0.045 | | |
| 3 | 0.032 | 55.3 | 0 |
| 2 | 0.049 | 14.9 | 0 |
| 2 | 0.069 | 24.7 | 0 |
| 2 | 0.057 | 36 | 0 |
| 2 | 0.039 | | |
| 3 | 0.051 | 60.5 | 0 |
| 4 | 0.082 | 23.2 | 0 |
| 3 | 0.021 | 25.1 | 1 |
| 1 | 0.05 | 84.3 | 0 |
| 2 | 0.042 | 74.4 | 0 |
| 1 | 0.042 | 85.1 | 0 |
| 3 | 0.04 | 12.4 | 0 |
| 3 | 0.023 | 30.2 | 1 |
| 2 | 0.043 | 62.2 | 0 |
| 2 | 0.036 | 36 | 0 |
| 2 | 0.038 | 43.7 | 0 |
| 3 | 0.052 | | |
| 3 | 0.048 | | |
| 2 | 0.046 | 8.6 | 1 |
| 3 | 0.021 | | |
| 3 | 0.05 | 43.5 | 0 |
| 3 | 0.044 | 42.6 | 0 |
| 1 | 0.06 | 84.7 | 0 |
| 3 | 0.03 | 8 | 1 |
| 2 | 0.045 | 83.4 | 0 |
| 2 | 0.047 | 79.5 | 0 |
| 3 | 0.035 | 66.6 | 0 |
| 3 | 0.054 | 66.6 | 0 |
| 2 | 0.056 | 81.2 | 0 |
| 2 | 0.029 | 70.8 | 0 |
| 3 | 0.041 | 28.4 | 1 |
| 3 | 0.068 | 5.1 | 1 |
| 3 | 0.026 | 63.3 | 0 |
| 3 | 0.049 | 56.6 | 0 |
| 2 | 0.034 | 48 | 0 |
| 2 | 0.073 | 8.2 | 1 |
| 3 | 0.031 | 11 | 0 |
| 3 | 0.055 | 37 | 1 |
| 3 | 0.074 | 35.3 | 0 |
| 2 | 0.073 | | |
| 2 | 0.02 | 38.6 | 0 |
| 2 | 0.04 | 16.8 | 1 |
| 2 | 0.047 | 78.9 | 0 |
| 2 | 0.038 | 67 | 0 |
| 2 | 0.038 | 22.8 | 0 |
| 4 | 0.078 | 20.7 | 1 |
| 2 | 0.046 | 43.5 | 0 |
| 2 | 0.048 | 72.5 | 0 |

| | | | |
|---|---|---|---|
| 2 | 0.049 | 28.6 | 0 |
| 2 | 0.043 | 24 | 0 |
| 1 | 0.049 | | |
| 2 | 0.049 | 69.2 | 0 |
| 3 | 0.133 | 58.9 | 0 |
| 2 | 0.063 | 67.3 | 0 |
| 1 | 0.079 | 67.4 | 0 |
| 3 | 0.061 | 14.8 | 1 |
| 2 | 0.044 | 51.8 | 1 |
| 3 | 0.061 | 64.8 | 0 |
| 2 | 0.052 | 12 | 0 |
| 4 | 0.096 | 20.1 | 1 |
| 3 | 0.044 | 21.2 | 1 |
| 3 | 0.045 | 19.4 | 1 |
| 4 | 0.07 | 36.3 | 1 |
| 2 | 0.048 | 64.6 | 0 |
| 3 | 0.05 | 66.8 | 0 |
| 2 | 0.03 | 12 | 0 |
| 2 | 0.039 | 59.1 | 0 |
| 2 | 0.056 | 62.6 | 0 |
| 3 | 0.079 | 12 | 0 |
| 2 | 0.069 | 84 | 1 |
| 4 | 0.052 | 26 | 1 |
| 1 | 0.036 | 64.5 | 0 |
| 3 | 0.064 | 21.5 | 1 |
| 3 | 0.035 | 13 | 1 |
| 1 | 0.048 | 57.3 | 0 |
| 4 | 0.094 | 19.6 | 0 |
| 1 | 0.058 | 64.1 | 0 |
| 3 | 0.058 | 64.3 | 0 |
| 4 | 0.078 | 32 | 1 |
| 2 | 0.032 | 31.3 | 0 |
| 2 | 0.027 | 53.3 | 0 |
| 4 | 0.089 | 36.1 | 1 |
| 2 | 0.063 | 58 | 0 |
| 4 | 0.071 | 15.9 | 1 |
| 3 | 0.029 | 56.2 | 0 |
| 1 | 0.055 | 56.9 | 0 |
| 3 | 0.033 | 53.3 | 0 |
| 2 | 0.044 | 52.6 | 0 |
| 4 | 0.096 | | |
| 2 | 0.037 | 53 | 0 |
| 4 | 0.1 | 48.2 | 0 |
| 2 | 0.036 | 22.3 | 1 |
| 2 | 0.044 | 53.3 | 0 |
| 2 | 0.033 | 43 | 0 |
| 4 | 0.054 | 13.6 | 1 |
| 4 | 0.069 | 10.9 | 1 |
| 3 | 0.07 | 44.5 | 0 |
| 1 | 0.061 | 0 | |
| 2 | 0.03 | 47.4 | 0 |
| 4 | 0.085 | 3.1 | 1 |
| 2 | 0.048 | 42.1 | 0 |
| 3 | 0.038 | 43.5 | 0 |

| | | | |
|---|---|---|---|
| 1 | 0.029 | 41.7 | 1 |
| 3 | 0.049 | 40.1 | 0 |
| 3 | 0.055 | 42 | 0 |
| 2 | 0.05 | 35 | 0 |
| 3 | 0.043 | 19.5 | 1 |
| 2 | 0.064 | 0 | |
| 3 | 0.058 | 0 | |
| 1 | 0.052 | 39.4 | 0 |
| 3 | 0.05 | 27 | 0 |
| 2 | 0.033 | | |
| 4 | 0.095 | 21 | 1 |
| 2 | 0.05 | 31.1 | 0 |
| 2 | 0.045 | 39 | 0 |
| 3 | 0.021 | 36.1 | 0 |
| 2 | 0.044 | 38.4 | 0 |
| 2 | 0.049 | 32.1 | 0 |
| 3 | 0.062 | 30.6 | 0 |
| 3 | 0.028 | 33.6 | 0 |
| 3 | 0.052 | 34.6 | 0 |
| 4 | 0.102 | 14.7 | 1 |
| 4 | 0.052 | 22.3 | 1 |
| 3 | 0.048 | 11.3 | 1 |
| 1 | 0.051 | 0 | |
| 3 | 0.054 | 0 | |
| 3 | 0.056 | 0 | |
| 1 | 0.057 | 0 | |
| 2 | 0.056 | 0 | |
| 3 | 0.046 | 0 | |
| 2 | 0.057 | 0 | |
| 3 | 0.055 | 17.7 | 1 |
| 3 | 0.052 | 0 | |
| 2 | 0.016 | 0 | |
| 2 | 0.083 | 0 | |
| 3 | 0.045 | 0 | |
| 2 | 0.062 | 0 | |
| 3 | 0.034 | 30.6 | 0 |
| 3 | 0.052 | 0 | |
| 1 | 0.055 | 0 | |
| 3 | 0.035 | 0 | |
| 2 | 0.067 | 0 | |
| 3 | 0.044 | 29.9 | 0 |
| 1 | 0.014 | | 0 |
| 2 | 0.04 | | |
| 3 | 0.033 | | |
| 4 | 0.079 | | |
| 2 | 0.017 | 28.3 | 0 |
| 2 | 0.046 | | |
| 3 | 0.024 | | |
| 1 | 0.046 | | |
| 3 | 0.035 | | |
| 1 | 0.036 | | |
| 4 | 0.198 | | |
| 3 | 0.044 | | |
| 3 | 0.064 | | |

| | | | |
|---|---|---|---|
| 3 | 0.027 | 12 | 0 |
| 4 | 0.092 | | |
| 3 | 0.053 | | |
| 4 | 0.084 | 22 | 1 |
| 1 | 0.054 | | |
| 2 | 0.037 | | |
| 1 | 0.05 | | |
| 3 | 0.034 | 28 | 0 |
| 2 | 0.046 | | |
| 2 | 0.049 | | |
| 4 | 0.06 | 8 | 1 |
| 1 | 0.051 | 19 | 0 |
| 3 | 0.065 | 20.5 | 0 |
| 3 | 0.041 | 16 | 0 |
| 4 | 0.068 | 20 | 0 |
| 3 | 0.06 | 20.4 | 0 |
| 3 | 0.08 | 28 | 0 |
| 2 | 0.032 | 15.6 | 0 |
| 3 | 0.061 | 15 | 0 |
| 4 | 0.064 | 11 | 1 |
| 4 | 0.11 | 15 | 1 |
| 2 | 0.037 | 15 | 0 |
| 2 | 0.043 | 16 | 0 |
| 3 | 0.031 | 17 | 0 |
| 2 | 0.054 | 12.5 | 0 |
| 3 | 0.062 | 14 | 0 |
| 2 | 0.05 | 14.1 | 0 |
| 2 | 0.067 | 13.3 | 0 |
| 4 | 0.105 | 13.3 | 0 |
| 1 | 0.046 | 11.6 | 0 |
| 2 | 0.048 | 11.9 | 0 |
| 4 | 0.056 | 84 | 1 |
| 4 | 0.066 | 12 | 0 |
| 4 | 0.037 | 10.1 | 1 |
| 3 | 0.051 | 84 | 1 |
| 3 | 0.042 | 67 | 1 |
| 4 | 0.052 | 48 | 1 |
| 3 | 0.053 | 40 | 1 |
| 4 | 0.071 | 38 | 1 |
| 4 | 0.036 | 36 | 1 |
| 3 | 0.071 | 24 | 1 |
| 3 | 0.059 | 24 | 1 |
| 3 | 0.041 | 25 | 1 |
| 3 | 0.051 | 22 | 1 |
| 3 | 0.052 | 24 | 1 |
| 3 | 0.046 | 24 | 1 |
| 3 | 0.052 | 17 | 1 |
| 3 | 0.053 | 13 | 1 |
| 3 | 0.067 | 11 | 1 |
